# The Medical Necessity for Medicinal Cannabis: Prospective, Observational Study Evaluating the Treatment in Cancer Patients on Supportive or Palliative Care

**DOI:** 10.1155/2013/510392

**Published:** 2013-07-16

**Authors:** Gil Bar-Sela, Marina Vorobeichik, Saher Drawsheh, Anat Omer, Victoria Goldberg, Ella Muller

**Affiliations:** ^1^Division of Oncology, Integrated Oncology and Palliative Care Unit, Rambam Health Care Campus, 31096 Haifa, Israel; ^2^Faculty of Medicine, Technion-Israel Institute of Technology, 31096 Haifa, Israel

## Abstract

*Background*. Cancer patients using cannabis report better influence from the plant extract than from synthetic products. However, almost all the research conducted to date has been performed with synthetic products. We followed patients with a medicinal cannabis license to evaluate the advantages and side effects of using cannabis by cancer patients. *Methods*. The study included two interviews based on questionnaires regarding symptoms and side effects, the first held on the day the license was issued and the second 6–8 weeks later. Cancer symptoms and cannabis side effects were documented on scales from 0 to 4 following the CTCAE. The distress thermometer was used also. *Results*. Of the 211 patients who had a first interview, only 131 had the second interview, 25 of whom stopped treatment after less than a week. All cancer or anticancer treatment-related symptoms showed significant improvement (*P* < 0.001). No significant side effects except for memory lessening in patients with prolonged cannabis use (*P* = 0.002) were noted. *Conclusion.* The positive effects of cannabis on various cancer-related symptoms are tempered by reliance on self-reporting for many of the variables. Although studies with a control group are missing, the improvement in symptoms should push the use of cannabis in palliative treatment of oncology patients.

## 1. Introduction

*Cannabis sativa* is one of the most ancient psychotropic drugs known to humanity. Evidence of the use of cannabis for medicinal and ceremonial purposes goes back 4000 years. In 1854, the plant appeared in the United States Dispensatory and was sold freely in pharmacies in Western countries. It also appeared in the British Pharmacopoeia as an extract and tincture for over 100 years. In 1942, cannabis was removed from the United States Pharmacopoeia, and, with that, its legal medicinal use was stopped. Only in 1971 did Britain and most of the European countries outlaw the use of cannabis according to the UN Convention of Psychotropic Substances [1]. Regardless, a rising number of states in the USA, Canada, and several European countries allow medicinal use of cannabis subject to a doctor's recommendation.

More than 60 C terpenophenols derived differently from one another in their chemical structure by location and number of carbons have been isolated from *Cannabis sativa* [2]. In the early 1990s, the endocannabinoid derivatives of arachidonic acid, which bind to cannabinoid receptors in the body, were located. The endocannabinoids are active mostly on the central nervous system as neuromodulators or retrograde messengers, which inhibit the release of other neurotransmitters [3], giving the background for the different unspecific influences of *Cannabis sativa* on different symptoms. Along with the popularity of the cannabis plant as an effective treatment for disease symptoms in oncology patients and for various medicinal indications unrelated to cancer patients, there is growing evidence to demonstrate interest in the use of cannabinoids in medicine, although high quality studies are still missing. There is a basic difficulty in conducting randomized, double-blind statistical power research in products that are extracted from plants. This difficulty arises from a lack of a driving economic source and from a difficulty in reaching set standards regarding the product and its quality over time, the method of consumption, and diversity of the population. As a result, although the users of cannabis report a better influence from the plant's extract as opposed to synthetic products, all the research conducted to this point has been performed with one of the three synthetic products that have gained approval from American, Canadian, or European international authorities.

In Israel, according to the Ministry of Health regulations, permission to use medicinal cannabis for oncology patients can be given for two indications: to relieve disease-related symptoms in advanced disease or during chemotherapy treatment in order to reduce side effects. The indications are very wide and allow a great deal of freedom for the physician's decisions but also cause high demands for cannabis from patients. The physician's recommendations for medicinal cannabis are referred to a few medical oncologists in the central governmental hospitals that have authorization from the Ministry of Health to issue a license to oncology patients for using medicinal cannabis.

Eight farms have Ministry of Health permission to grow cannabis for medicinal use, and four companies have permission to deliver the cannabis to the patients. The cannabis grown on the farms is a cultivated species, a combination of *Cannabis sativa *and* Cannabis indica* that differs from 70% *indica* and 30% *sativa *to 90% *sativa* and 10% *indica.* The concentrations of the main cannabinoid differ between the species. The delta-9-tetrahydrocannabinol (Δ9-THC) concentrations are usually between 16% and 27% and the cannabidiol (CBD) concentrations between 0.5% and 2%. The patient's license allows 30 grams per month. Patients are advised to start with a species with less than 20% Δ9-THC in the first two months and to begin with a low use, slowly increasing the daily use according to the needs and side effects.

The current study followed prospectively consecutive patients who received a medicinal cannabis license at Rambam Health Care Campus in Haifa, Israel, in order to evaluate the advantage, side effects, and administrative problems concerning the daily use of cannabis in cancer patients.

## 2. Patients and Methods

### 2.1. Sample and Procedure

According to the regulations in Israel for the use of medicinal cannabis, after approval of the license by the regulator physician, patients must receive guidance on the correct way to start the treatment and possible side effects. The guidance sessions are given personally to the patients by nurses who underwent a course conducted with the permission of the Ministry of Health on cannabis use.

After approval of the study protocol by the institutional ethics committee, patients who came for the guidance sessions before the license was issued were asked to participate in the study. Between January 2011 and March 2012, 244 new licenses were issued to oncology patients, 211 of whom signed the informed consent and entered the study. The study included two interviews by the nurses who gave guidance on cannabis use. The interviews were based on questionnaires testing the level of the symptoms and possible side effects of cannabis, together with demographic details and other medications. The first interview was held on the day of the guidance session and the second by telephone 6–8 weeks later by the same nurse. Data on the illness, the oncology treatment, and the reasons for cannabis use were also collected from the records sent by the treating oncologist/hematologist or palliative physician to the regulator physician.

### 2.2. Interview Questions

The data from the treating physician's recommendation for cannabis included basic demographic data, cancer diagnosis, active oncology treatment or best supportive care only, aim of the oncology treatment (curative or palliative), reason for recommendation to use cannabis, past treatments for the symptoms that are the reason for the cannabis recommendation, and the preferred way of using the cannabis (smoking, inhalation, oil).

The interview on day one added details to the demographic data, such as education, past smoking of tobacco or cannabis, resources of recommendations to use cannabis, reasons for asking for a cannabis prescription, chronic medications and specific pain medications, opioids, antidepressants or antianxiety medications, and steroids. Information on cancer symptoms or chemotherapy-related side effects that could be influenced by the cannabis included nausea, vomiting, mood disorders, fatigue, weight loss, anorexia, constipation, sexual function, sleep disorders, itching, and pain, neuropathic or nociceptive. Symptoms that could be related later to cannabis side effects were also documented, including infections needing antibiotics in the previous six weeks, mouth dryness, cough, shortness of breath, diarrhea, dizziness, loss of coordination, and memory loss. Data on leukocyte count and albumin level were taken from the last documented blood tests.

The second interview was held by telephone 6–8 weeks after the first interview. The second interview was not conducted personally due to concerns that some patients would not come and it would cause bias if some of the second interviews were made by telephone and some personally.

The data collected included information on the cannabis use, such as regular use or interrupted. If the treatment stopped, the reasons for stopping were documented. Patients who continued the treatment were asked again about cancer symptoms or chemotherapy-related side effects and possible cannabis side effects.

The cancer symptoms and the cannabis side effects were documented on scales from 0 to 4 following the Common Terminology Criteria for Adverse Events (CTCAE), version 4.03 [4]. Cancer-related symptoms and/or oncology treatment side effects were calculated as a score of the sum of all the grading scores of the different symptoms and nominate-symptom score. The distress thermometer was used as a basic tool to evaluate the current symptomatic status of the patients on the day of the interview. This easy to use tool has been validated in many studies as a clinical tool for evaluation of the need for symptomatic relief intervention [5].

### 2.3. Statistics

Differences in patient characteristics and comparisons between groups of continuous cannabis use and early determination were detected by the chi-square test. Changes in the degree of cancer-related symptoms or oncology treatment side effects and possible side effects of cannabis use in the patients with prolonged use were also evaluated by the chi-square test. The differences in the symptom sums between patient characteristics subgroups according to the different interviews were calculated with Mann-Whitney nonparametric tests in the condition of two subgroups or analysis of variance (Kruskal-Wallis) test for three or more subgroups, with Bonferroni post hoc multiple comparisons. Wilcoxon two related simple nonparametric test was used to test the change in symptom sum between the two time points of the interviews. Interactions between subgroups of patient characteristic and the change of the symptoms sum by time were indicated by the repeated measures analysis. *P* value and estimate of effect size (eta) were calculated. A two-tailed *P* value of 0.05 or less was considered as statistically significant. Statistical analyses were performed with SPSS (Statistics Products Solutions Services) 18.0 software for Windows.

## 3. Results

### 3.1. Study Population

Two hundred and eleven patients signed the informed consent and had the first interview on the day of receiving the cannabis license. Of these, 106 (50%) patients continued the treatment for a long period and had a second interview by telephone. Fifty (24%) patients died in the period before the second interview, and 10 (5%) patients were lost to follow-up (no answer to the telephone in three calls at different hours and days). Twenty (10%) patients did not start the cannabis treatment, although they had a license. The main reasons, as given on the telephone in the second interview, were fear of being perceived as a criminal, fear of starting to smoke again, general improvement due to anticancer treatment, or symptomatic improvement due to other symptomatic-related drugs. Twenty-five (12%) patients stopped treatment after less than a week, mainly because of the side effects that influenced their quality of life or the absence of any clinical improvement. The side effects that caused early determination of cannabis included acute psychosis, anger attacks, dizziness, somnolence, fainting events, burning throat, nausea and vomiting, and test changes.

Patient characteristics and demographic data, including comparison between the groups of continuous treatment to early determination, are presented in Table 1. The patient group that stopped cannabis use early had significant more CNS involvement (*P* = 0.006), less chemotherapy-induced nausea and vomiting (*P* = 0.002), and less anorexia or weight loss (*P* = 0.007) according to their referring physicians' reports. Also, according to the physician recommendation for cannabis use, fewer patients seek cannabis due to previous supportive treatments not being satisfactory (*P* < 0.001).

### 3.2. Symptom Control by Cannabis in the Group with Continuous Treatment

Cancer symptoms, anticancer treatment-related side effects, and possible cannabis side effects were evaluated in the two interviews. Although the 106 patients who continued cannabis use are a selected bias group, this biased group represents the regular medicinal cannabis users. All cancer or anti-cancer treatment-related symptoms, including nausea, vomiting, mood disorders, fatigue, weight loss, anorexia, constipation, sexual function, sleep disorders, itching, and pain had significant improvement (*P* < 0.001) (Table 2, using distribution to three groups, without the symptom (grade 0), minimal (grade 1 or 2), or severe disturbance to daily life (grade 3 or 4)). Thirty-one (29%) patients had a symptom score ≤10, and 22 (21%) patients had ≥17 score before cannabis use, compared to 71 (71%) patients with ≤10 score and 8 (8%) patients with ≥17 score (*P* < 0.001) in the second interview.

To add objective parameters to the patients' self-reports during the interviews, changes in pain or depression/anxiety drugs were recorded as well. Seventy patients used pain medication at the beginning, of whom 2 (1.7%) had dose elevation and 31 (43%) dose reduction. Six of 36 (17%) patients who were without treatment started antipain medications. Twenty-one patients used anti-depression/anxiety drugs, of whom 7 (33%) reduced the dose and one started new medication. Among the other 85 patients, only 5 (5.8%) started new medication.

There were no significant side effects to the cannabis except for memory lessening in the 106 patients who continued cannabis use. No significant difference was found in the level of infections, mouth dryness, cough, shortness of breath, diarrhea, and leukocyte count or albumin level during the time between the two interviews.

Regarding memory lessening, in the first interview, 83 (78%) patients reported no interferences in memory. In the second interview, only 66 (62%) patients reported no interferences in memory (*P* = 0.002), and all others reported memory lessening in different degrees. The number of patients with memory lessening varies according to age, with 15.8% of the patients younger than 31 years compared to 25.2% of older ages; use of antidepressant or antianxiety drugs, 34% compared to 17% of nonusers; and past cannabis users 38% compared to 18.2% of non-users. These differences were not statistically significant.

### 3.3. Degree of Symptomatic Relief Related to Patient Characteristics in the Group with Continuous Treatment

For the group of patients who continued cannabis use, the symptom score was improved in 32.1% and worsened in 3.8% of patients, with a mean score of 13 (range, 4–30) before treatment and 7 (range, 7–20) in the second interview.  Table 3 summarizes the degree of symptomatic relief related to patient characteristics. At the first interview, there were a few parameters with significant differences in symptom scores between the sub-groups: radiotherapy in the previous 3 months (*P* = 0.03), mood disorders (*P* = 0.001), weight loss (*P* = 0.02), memory loss (*P* = 0.049), and distress thermometer (*P* = 0.002). At the second interview, no significant difference between the sub-groups was seen, although a trend of keeping the difference in symptom score was still seen in past cannabis smoking (*P* = 0.057), distress thermometer (*P* = 0.055) and oncology treatment aim; curative/adjuvant or palliative (*P* = 0.052). In all parameters, the difference in symptom score showed a direction of improvement in all sub-group analyses (*P* < 0.001). Interaction was found according to age (*P* = 0.004, eta −0.06), depression or anxiety level (*P* = 0.002, eta −0.14) and degree of memory loss (*P* = 0.004, eta −0.09).

## 4. Discussion

In the current study population, 25% of the patients who entered the study died after a short period of time, less than two months. In the group that continued the cannabis use, it was highly effective for many symptoms connected to advanced disease, which raises the question of whether physicians are prescribing the cannabis too late, and if so, why? A study carried out among hospice health care providers in the USA, using a questionnaire to assess knowledge, experience, and views regarding the use of cannabis in terminally ill patients, revealed a generally favorable approach to legalization of cannabis and its use in symptom management for their terminally ill patients [6]. Severe side effects of cannabis or ineffectiveness together with mild side effects caused early determination of treatment in 12% of the patients. This patient group had more CNS involvement but, nevertheless, was less symptomatic than the continued cannabis users. The side effects reported by both groups who used cannabis were not different from the known side effects of cannabis in noncancer patients. Although the characteristic differences between the groups were not significant, it seems that the patients who continued the treatment were more symptomatic at the beginning, according to their reports and their physicians' reports in the records for cannabis licenses. In the current study, 29% of the patients did not have the second interview, and another 21% did not give information about the influence of continuous cannabis use. Therefore, the results of the second interview should be taken with caution.

The cannabis plant and the synthetic drugs based on the plant are considered medically safe. Most of the adverse effects are related to the fact that the plant and the drugs are psychoactive, mostly depending on their concentration and on the Δ9-THC dosage. Among the effects named were dizziness, euphoria, difficulty concentrating, disturbances in thinking, memory loss, and loss of coordination [7].

Memory lessening was the only significant reported side effect among the prolonged users. Neuropsychological decline is well known to result from prolonged time and persistent cannabis use, as was reported in the Dunedin Study, for example, a prospective study of a birth cohort of individuals followed from birth to age of 38 years [8]. Also in the short term, cannabis use can cause a modest effect on self-reported problems with prospective memory, with a primary problem with retrospective memory appearing to underlie this relationship, as was found in a prospective study with healthy individuals [9]. The significant memory lessening in the current study may be related to additive or synergistic influences of comedications, such as opioids and antidepressive drugs, or to the older age of most of the users, compared to the usual study population of cannabis users. Further research to clarify this point is needed.

The population of the prolonged users in the current study reported significant improvement in all aspects of supportive and palliative oncology care. This group of patients represents unselected symptomatic cancer patients, and the results are mainly based on patients self-reports during the interviews. The results from prospective randomized trials on different cannabinoids were less significant. Clinical trials conducted on the effect of medicinal cannabis on pain reduction in cancer patients were either negative or inconclusive. It should be noted that these research projects included a small number of participants, and some were observational [2, 10]. Recently, a randomized, double-blind, placebo-controlled, graded-dose study in patients with advanced cancer and opioid-refractory pain receiving placebo or nabiximols showed efficacy in reducing the average daily pain from baseline and reduction in sleep disruptions. Those effects were seen mainly in the groups treated with low and medium doses [11].

Interesting, although not with cancer patients, is the double-blind, placebo-controlled, crossover study evaluating the analgesic efficacy of vaporized cannabis in subjects who experienced neuropathic pain despite traditional treatment. This study included 39 patients with central and peripheral neuropathic pain. The vaporized cannabis, even at low doses, showed analgesic efficacy with minimal psychoactive effects and may present an effective option for patients with treatment-resistant neuropathic pain [12]. In the current study, the number of patients with severe pain (grade 3-4) was cut by half. Patients with neuropathic or nociceptive pain were improved.

Research with cannabinoids for the prevention of nausea and vomiting in patients receiving chemotherapy was conducted in the 1980s toward the approval of these drugs for this indication, compared to placebo or various neuroleptic drugs. A meta-analysis that combined the different cannabis treatments compared to neuroleptics and included over 1100 patients from 18 studies found a strong significance in favor of the cannabinoids [13]. It should be noted that these drugs were not compared to any of the new antinausea drugs. According to the interviews in this study, cannabis has an additional advantage, mainly in persistent nausea or vomiting in addition to the new medications for this indication.

Another indication for cannabis treatment in cancer patients is loss of appetite and weight loss (anorexia and cachexia). Although positive results were reported among AIDS patients, two studies in cancer patients were negative. A random study compared administration of dronabinol to megestrol acetate to both together [14], and the second study compared the administration of a combination of Δ9-THC and CBD to Δ9-THC alone, compared to placebo [15].

Both symptom score and distress thermometer showed direction of improvement in all subgroups. The strongest interaction was found to be with depression or anxiety level at the first interview. The large improvement in physical symptoms can be partially related to the cannabis-induced euphoria, but, from a medical point of view, the general improvement in the level of distress is important as an end-point for palliative studies [16], and the cause is less important. A slight reduction in the need for opioids and antidepressant drugs was seen in the study group. In a study that followed the trends of legal medicinal cannabis users in The Netherlands, the level of morphine use was low but stable. The use of nonopioid analgesics and antidepressants was very fluctuating during the survey period [17].

Although the daily dose of the cannabis was constant, the patients who entered the study used different cannabis species with different concentrations of the main cannabinoid that came from different cannabis farms. This could influence the effects of the treatment and the side effects but is part of the daily practice of the medicinal use of cannabis. It is important to note that a dose-limiting factor is how much Δ9-THC may be tolerated. In two small studies that tested the influence of smoking high-dose, low-dose, or placebo cannabis [18] or inhaling medium-dose, low-dose, or placebo cannabis [19] on neuropathic pain, positive results were reported. Nevertheless, cognitive effects become more pronounced according to the cannabis dose and especially in cannabis naïve individuals.

The current study is observational and has some limitations, mainly the lack of an appropriate control group for comparison, since the overall improvement in perceived health quality might be attributed simply to time or other factors unrelated to treatment. Also, the fact that cannabis effects are only reported when self-report-based methodologies are used may imply that the “real” effect may be one of psychological order, rather than specific effects on the body physiology.

## 5. Conclusion

The positive effects of cannabis on various cancer-related symptoms are tempered by reliance on self-reporting for many of the variables, as was done with the limitation of an observational study. Although studies with a control group are missing, the improvement in symptoms should push the use of cannabis in the practice of oncology palliative treatment.

## Figures and Tables

**Table 1:** Characteristics and demographics of patients included in the study, according to their referring physicians' reports and comparing the groups of continuous treatment to early determination.

	All patients	Continuous cannabis use	Early determination	*P* value
No. of patients	211	106	25	
Age:				
≤30 years 31–70 years ≥71 years	29 (14%) 117 (55%) 65 (31%)	19 (18%) 58 (54%) 29 (28%)	1 (4%) 15 (60%) 9 (36%)	0.12
Gender:				
Male Female	126 (60%) 85 (40%)	65 (59%) 45 (41%)	11 (44%) 14 (56%)	0.18
Academic studies	108 (51%)	61 (56%)	15 (60%)	0.98
Reasons for cannabis use (physician's recommendation)				
Previous treatment not satisfactory Others	152 (72%) 59 (28%)	94 (87%) 12 (13%)	11 (44%) 14 (56%)	<0.001
Primary tumor:				
Lung Breast Gastrointestinal Hematological Other	45 (21%) 20 (9%) 55 (27%) 27 (12%) 54 (31%)	18 (17%) 12 (11%) 36 (34%) 16 (15%) 22 (23%)	7 (28%) 3 (12%) 4 (16%) 2 (8%) 9 (36%)	0.2
CNS involvement	20 (9%)	5 (5%)	6 (24%)	0.006
Oncology treatment aim:				
Curative/adjuvant Palliative	99 (47%) 112 (53%)	56 (53%) 50 (47%)	13 (52%) 12 (48%)	0.99
Chemotherapy during last 3 months	156 (74%)	81 (75%)	18 (72%)	0.80
Radiotherapy during last 3 months	51 (24%)	23 (21%)	7 (28%)	0.59
Chemotherapy-induced nausea and vomiting	48 (23%)	33 (31%)	2 (8%)	0.002
Fatigue	93 (44%)	46 (43%)	9 (36%)	0.65
Anorexia/weight loss	104 (49%)	59 (55%)	6 (24%)	0.007
Depression/anxiety	72 (34%)	40 (37%)	7 (28%)	0.49
Opioid use	110 (52%)	60 (56%)	12 (48%)	0.51
Anti-depressant/anxiety use	76 (36%)	44 (42%)	6 (24%)	0.34
Steroid use	96 (45%)	49 (45%)	9 (36%)	0.50

**Table 2:** Changes in degree of cancer-related symptoms or oncology treatment side effects among patients with continuous cannabis use.

	Grade	First interview	Second interview
Nausea	0	34 (32%)	73 (69%)
	1-2	69 (65%)	29 (27%)
	3-4	3 (3%)	4 (4%)
Vomiting	0	73 (69%)	98 (92%)
	1-2	33 (31%)	8 (8%)
Mood disorders	0	6 (6%)	48 (46%)
	1-2	79 (74%)	49 (46%)
	3	21 (20%)	9 (8%)
Fatigue	0	3 (3%)	48 (45%)
	1-2	47 (44%)	53 (50%)
	3-4	56 (53%)	5 (5%)
Weight loss	0	35 (33%)	71 (67%)
	1-2	66 (62%)	35 (33%)
	3	5 (5%)	0
Anorexia	0	34 (32%)	72 (68%)
	1-2	69 (65%)	29 (27%)
	3-4	3 (3%)	5 (5%)
Constipation	0	45 (42%)	71 (67%)
	1-2	54 (51%)	32 (30%)
	3	7 (7%)	3 (3%)
Sexual function	0	26 (25%)	51 (48%)
	1-2	30 (28%)	18 (17%)
	3	50 (47%)	37 (35%)
Sleep disorders	0	21 (20%)	59 (56%)
	1-2	74 (70%)	38 (36%)
	3-4	11 (10%)	9 (8%)
Itching	0	72 (68%)	90 (85%)
	1-2	30 (28%)	15 (14%)
	3	4 (4%)	1 (1%)
Pain	0	20 (19%)	44 (42%)
	1-2	32 (31%)	36 (34%)
	3-4	54 (51%)	26 (25%)

**Table 3:** Association between patient characteristics with cancer-related symptoms among long-term cannabis users.

	No. of patients	Symptom score 1st interview (median, range)	Symptom score 2nd interview (median, range)	Difference in symptoms score between groups, before treatment	Difference in symptoms score between groups, after treatment	*P* value
All patients	106	13 (4–30)	7 (0–20)			<0.001
Age:						
≤30 years 31–70 years ≥71 years	19 58 29	11 (6–30) 13 (4–24) 13 (6–22)	4 (1–17) 7 (0–20) 8 (1–19)	0.88	0.06	<0.001 <0.001 <0.001
Gender:						
Male	63	12 (6–30)	7 (0–20)	0.95	0.98	<0.001
Female	43	14 (4–21)	7 (1–19)			
Oncology treatment aim:						
Curative/adjuvant Palliative	57 49	11 (6–22) 13 (4–30)	5 (0–18) 7 (0–20)	0.15	0.052	<0.001 <0.001
Chemotherapy last 3 months:						
Yes No	79 27	13 (4–30) 11 (6–24)	7 (0–20) 7 (0–18)	0.09	0.89	<0.001 <0.001
Radiotherapy last 3 months:						
Yes No	22 84	15 (6–24) 12 (4–30)	9 (1–19) 7 (0–20)	0.03	0.2	<0.001 <0.001
Past cannabis smoking:						
Yes No	29 77	12 (6–30) 13 (4–22)	5 (1–20) 8 (0–19)	0.3	0.057	<0.001 <0.001
Cigarette smoking:						
Yes No In the past	25 30 51	13 (4–22) 13 (6–21) 13 (6–30)	8 (1–19) 6 (0–20) 9 (0–18)	0.35	0.29	<0.001 <0.001 <0.001
Mood disorders:						
No Low Severe	6 79 21	13 (8–20) 12 (4–24) 16 (4–30)	10 (4–16) 6 (0–20) 9 (2–19)	0.001	0.06	<0.001 <0.001 <0.001
Weight loss:						
Yes No	58 48	14 (4–30) 11 (6–20)	8 (0–19) 6 (1–20)	0.02	0.1	<0.001 <0.001
Memory loss:						
Yes No	23 83	14 (6–30) 12 (4–24)	8 (0–19) 7 (0–20)	0.049	0.18	<0.001 <0.001
Pain:						
Neuropathic Nociceptive	24 56	14 (6–30) 13 (6–24)	7 (2–19) 8 (0–20)	0.38	0.71	<0.001 <0.001
Distress thermometer:						
1–4 5–7 8–10	34 36 36	12 (6–21) 12 (4–24) 16 (6–30)	6 (1–16) 7 (1–20) 9 (0–19)	0.002	0.055	<0.001 <0.001 <0.001
Symptom score:						
4–10 11–16 ≥17	31 53 22	8 (4–10) 13 (11–16) 20 (17–30)	4 (1–20) 7 (0–19) 12 (3–19)		<0.001	<0.001 <0.001 <0.001
